# Pain Relief after Surgical Decompression of the Distal Brachial Plexus

**DOI:** 10.1055/s-0040-1716718

**Published:** 2020-10-16

**Authors:** Richard Morgan, Iain Elliot, Vibhu Banala, Christopher Dy, Briana Harris, Elizabeth Anne Ouellette

**Affiliations:** 1 Department of Physical Medicine & Rehabilitation, Larkin Community Hospital, Miami, Florida, United States; 2 Department of Orthopedics and Sports Medicine, University of Washington, Seattle, Washington, United States; 3 Department of Orthopedic Surgery, Montefiore Medical Center, Bronx, New York, United States; 4 Department of Orthopedic Surgery, Washington University, School of Medicine, St. Louis, Missouri, United States; 5 Department of Orthopedic Surgery, Miami Orthopedics and Sports Medicine Institute, Baptist Health Medical Group South Florida, Miami, Florida, United States

**Keywords:** brachial plexus, brachial plexopathy, medial brachial fascial compartment, outcomes, surgery, pain, entrapment, compression, neuropathy

## Abstract

**Background** Brachial plexopathy causes pain and loss of function in the affected extremity. Entrapment of the brachial plexus terminal branches within the surrounding connective tissue, or medial brachial fascial compartment, may manifest in debilitating symptoms. Open fasciotomy and external neurolysis of the neurovascular bundle in the medial brachial fascial compartment were performed as a surgical treatment for pain and functional decline in the upper extremity. The aim of this study was to evaluate pain outcomes after surgery in patients diagnosed with brachial plexopathy.

**Methods** We identified 21 patients who met inclusion criteria. Documents dated between 2005 and 2019 were reviewed from electronic medical records. Chart review was conducted to collect data on visual analog scale (VAS) for pain, Semmes-Weinstein monofilament test (SWMT), and Medical Research Council (MRC) scale for muscle strength. Pre- and postoperative data was obtained. A paired sample *t* -test was used to determine statistical significance of pain outcomes.

**Results** Pain severity in the affected arm was significantly reduced after surgery (pre: 6.4 ± 2.5; post: 2.0 ± 2.5; *p* < 0.01). Additionally, there was an increased response to SWMT after the procedure. More patients achieved an MRC rating score ≥3 and ≥4 in elbow flexion after surgery. This may be indicative of improved sensory and motor function.

**Conclusion** Open fasciotomy and external neurolysis at the medial brachial fascial compartment is an effective treatment for pain when nerve continuity is preserved. These benefits were evident in patients with a prolonged duration elapsed since injury onset.

## Introduction

The brachial plexus is a complex network of nerves that transmits motor and sensory signals responsible for function of the upper extremity. Injury to the brachial plexus, known as brachial plexopathy, is most often a consequence of trauma. 1 2 It is well known that the functional impairments associated with brachial plexopathy hinder dexterity and performance of daily routines leading to disability. In addition to aberrant extremity function, a significant individual predictor of disability severity is pain. 3 4 Indeed, approximately 55 to 95% of brachial plexus injured patients endure neuropathic pain within the affected extremity. 5 6 7 In many cases, pain is a deterrent against extremity movement. As a consequence, adherence to rehabilitative modalities requiring use of the affected limb is jeopardized because of the persistent fear of pain exacerbation. 8 Pain associated with brachial plexopathy poses a burden to the overall quality of life of patients and clinical management by clinicians. 9 10 11 12

Brachial plexopathy is a complex and heterogenous condition that necessitates the use of a substantial amount of resources and a broad array of treatments for each unique patient. Many variables must be considered in the assessment and treatment of brachial plexopathy. These include but are not limited to patient demographics, mechanism of injury, comorbid injuries and medical conditions, severity and location of nerve lesion(s) as well as extent of plexus damage. 13 14 15 The conservative approach to neuropathic pain often entails “trial and error” of different treatment modalities. Medications used for neuropathic pain often have unpredictable efficacy, decreased benefits over time, and carry the risk of side effects. The utility of physical and occupational therapy, osteopathic manipulative treatment, massage therapy, and acupuncture may be limited due to pain.

Surgical interventions are generally reserved for cases refractory to conservative management. Advances in microsurgical techniques have spurred the development of techniques that effectively repair injured nerves. Notwithstanding, there remains continued debate regarding the diagnostic approach, timing of surgery, appropriate selection of surgical technique, and parameters for acceptable outcomes. 16 To enhance the value of surgery in brachial plexus injury, treatment objectives must be stratified and translated into outcome studies that encompass patient and surgeon-reported data.

Prior literature has revealed that only 19% of published articles on surgical techniques used in brachial plexopathy reported a pain outcome. 17 Most of these outcome studies have solely focused on motor function recovery. Overemphasis on a single parameter dismisses the global outcome assessment comprised of surgeon and patient perspectives. Objectives from the patient point of view may include return of independence, employment or school, preinjury lifestyle and social interactions as well as cosmesis, improved emotional well-being and pain relief. 9 14 17 18 Given the substantial impact of pain on overall quality of life, we sought to investigate pain outcomes when a surgical procedure more commonly used at other sites of nerve compression was employed in the brachial plexus.

We have identified an underreported source of neuropathic pain at the distal brachial plexus terminal branches. Several case reports have suggested that neuropathic pain can originate from compression of the terminal branches within the medial brachial fascial compartment (MBFC). 19 20 21 22 23 On the basis of these findings, a comprehensive investigation on the causes and management is warranted. Compression of nerves within the MBFC may gradually occur after trauma manifesting in delayed onset symptoms. 24 Thus, it is possible that the proportion of patients with brachial plexopathy related to polytrauma is greater long-term than 1.2% at initial assessment. 2 We believe that a progressive, low-grade, compression neuropathy develops within the MBFC after injury to the upper arm in many of these cases. Open fasciotomy of the brachial fascia and external neurolysis within the MBFC was performed in candidates who opted for surgical treatment.

The goal of the present study is to assess pain outcomes in patients diagnosed with brachial plexopathy who underwent open fasciotomy and external neurolysis at the MBFC. Our results demonstrate for the first time the effectiveness of this procedure at the MBFC in decreasing pain for patients diagnosed with nerve-in-continuity brachial plexus lesions.

## Patients and Methods

We conducted a retrospective chart review using the electronic medical records (EMR) at Baptist Hospital South Florida. Patients evaluated in clinic from 2005 to 2019 were identified by ICD-9 and ICD-10 codes in the EMR. The following ICD codes were required for inclusion: 953.4 (brachial plexus injury), 353 (brachial plexus lesions), G54.0 (brachial plexus disorders), and S14.3 (injury of the brachial plexus).

**Fig. 1:**
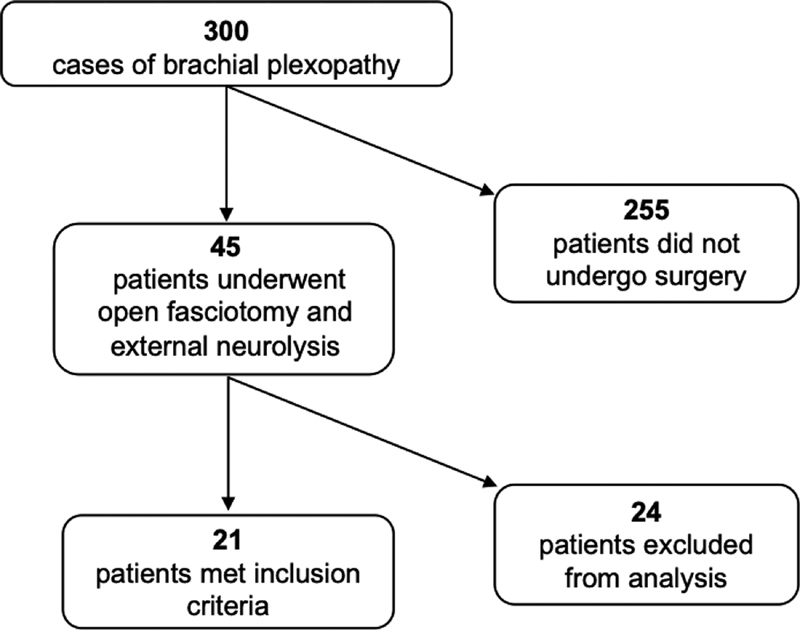
Study flow diagram.

Patients who fulfilled selection criteria were over the age of 18 years at the time of surgery, diagnosed with unilateral brachial plexopathy, and completed the present procedure. Patients who had obstetric complications, shoulder surgery, or other nerve repair procedures of the brachial plexus did not meet inclusion criteria. During chart review, we identified a total of 300 patients in our clinic who were given a diagnosis of brachial plexopathy. Of the 300 patients, 45 elected for surgical treatment and 21 met inclusion criteria for this study ( Fig. 1 ).

Data collection included demographic information, surgical history, etiology, side of injury, dominant arm, interval from symptoms onset to surgery and from surgery to postoperative evaluation (≥6 months), visual analog scale (VAS) pain scores, Semmes-Weinstein monofilament test (SWMT) scores, and Medical Research Council (MRC) motor strength grades. VAS pain scores for only the brachium were recorded. SWMT results from the finger pulps were noted. MRC rating scores were assessed for elbow flexion, finger abduction, proximal interphalangeal joint (PIP)/distal interphalangeal joint (DIP) extension, and thumb abduction. All data were ascertained from documents dated before and after surgery. A minimum of 6 months elapsed from surgery was required for data inclusion. There were no limits on duration of injury prior to surgery. Clinical findings and surgical procedures were completed by the senior author.

VAS and SWMT outcomes were analyzed for statistical significance using a paired sample *t* -test. *p* -Values <0.05 were considered to be statistically significant. Descriptive analysis was performed to assess demographic data and compare MRC rating grades. MRC grades ≥ 3 and ≥ 4 were used as parameters for muscle strength.

## Surgical Candidate Selection

Pain unresponsive to conventional management was the single most important indication for surgery. Most patients were referred by specialists for “last ditch” management of neuropathic symptoms. Brachial plexopathy originating at the MBFC is a clinical diagnosis. The surgical indications and contraindications used in our clinic are demonstrated in Tables 1 and 2 .

**Table 1:** Surgical indications

Indications		
History of present illness		
Pain	>3 mo	
	Progressively worsening, typically mild to moderate.	
	Originates in the brachium and extends to finders.	
	Brachium: sharp, gnawing or pressure sensation.	
	Distal pain typically variable in intensity and characterization.	
	Typically spares the shoulder.	
	Does not respond well to medications.	
Sensation	Numbness and tingling.	
	Involves the brachium, forearm, hand, and fingers.	
	Typically spares the shoulder.	
Muscle strength:	Progressive weakness	
	Difficulties performing activities of daily living without assistance from unaffected arm.	
	Weakness has impacted work performance.	
Physical examination		
Sensory	Tenderness to palpation over medial bicipital groove (i.e., positive Tinel's sign).	
	Abnormalities on SWMT.	
Motor	MRC grade < 5 in elbow flexion.	
	Abnormalities on motor exam not restricted by pain.	
Electrodiagnostic testing		
NCS	Slowing of conduction velocity.	
	Decreased amplitude	
EMG	Fibrillations	
	PSW	
	Reduced recruitment	

Abbreviations: EMG, electromyography; MRC, Medical Research Council; NCS, nerve conduction study; PSW, positive sharp wave; SWMT, Semmes-Weinstein Monofilament Test.

Note: This is not a definitive guide for surgery. Surgical consideration involves clinical judgment of the physician. Compression neuropathy within the brachial fascial compartment is a clinical diagnosis. It is a diagnosis of exclusion. The procedure must be thoroughly discussed with candidates. Discussion must include risks and benefits. Patients must express an understanding of all that is involved with the procedure.

**Table 2:** Surgical contraindications

Contraindications
• Patient does not have medical or cardiac clearance for surgery.
• Patients who have not attempted conservative management.
• Evidence of root avulsion, radiculopathy, or myelopathy.
• Diagnoses of brachial neuritis or plexitis, CRPS, thoracic outlet syndrome, or infection.

Abbreviation: CRPS, complex regional pain syndrome.

*Note:* This is not a definitive guide for surgery. Surgical consideration involves clinical judgment of the physician. Compression neuropathy within the brachial fascial compartment is a clinical diagnosis. It is a diagnosis of exclusion. The procedure must be thoroughly discussed with candidates. Discussion must include risks and benefits. Patients must express an understanding of all that is involved with the procedure.

All practical steps should be conducted to rule out neurological, vascular, inflammatory etiologies of the cervical spine, shoulder, and arm. The differential diagnoses included cervical radiculopathy, nerve root avulsion, pre- or post-ganglionic nerve rupture, thoracic outlet syndrome, Parsonage-Turner syndrome, complex regional pain syndrome, shoulder impingement, rotator cuff tear, and biceps tendinopathy. Compression neuropathy at the MBFC is a diagnosis of exclusion.

Electrodiagnostic studies were performed at third-party institutions as a continuation of clinical evaluation. Nerve conduction studies ( Table 3 ) and electromyography ( Table 4 ) demonstrated mixed abnormalities amongst surgical candidates. Abnormalities in conduction velocity, amplitude, fibrillation potentials, and positive sharp waves were observed. All electrodiagnostic reports were indicative of nerve-in-continuity brachial plexus lesions. Cubital tunnel syndrome and carpal tunnel syndrome were prevalent among surgical candidates. Release of distally entrapped nerves, as indicated from neurodiagnostic testing, was performed at the same time as the present procedure.

**Table 3:** Summary of preoperative nerve conduction studies

Function	Nerve (affected arm)	Stimulus site	Recording site	Latency (ms)	Amplitude (μV/mV)	Velocity (m/s)
Sensory	Median	Wrist	2nd digit	2.6 ± 0.5	16 ± 13.4	42.4 ± 7.4
	Ulnar	Above elbow	5th digit	2.3 ± 0.4	11.6 ± 8.6	46.7 ± 6.2
	Radial	Wrist	Base 1st digit	2 ± 0.4	11.1 ± 9.6	43.5 ± 10.3
Motor	Median	Wrist	Abd poll brev	4 ± 0.5	4.9 ± 2.4	43.8 ± 8.5
	Ulnar	Above elbow	Abd dig minimi	8.85 ± 1.15	4.6 ± 2.4	46.2 ± 10.4

Abbreviations: Abd dig minimi, abductor digiti minim; Abd poll brev, abductor pollicis brevis.

Note: Nerve conduction studies performed for motor (median and ulnar) and sensory (median, ulnar, and radial) nerves before surgery. Median of latency, amplitude, and velocity ± median absolute deviation (MAD); *N* = 21. Normal distribution violated (significant Shapiro-Wilk test).

**Table 4:** Summary of preoperative EMG studies

		Recruitment		Fibrillations		PSW	
Muscle	Normal	Decreased	Absent	Present	Absent	Present	Absent
First dorsal interosseous	14 (67%)	7 (33%)	0 (0%)	8 (38%)	13 (62%)	7 (33%)	14 (67%)
Abductor pollicis brevis	14 (67%)	7 (33%)	0 (0%)	6 (29%)	15 (71%)	4 (19%)	17 (81%)
Biceps	13 (62%)	8 (38%)	0 (0%)	4 (19%)	18 (86%)	4 (19%)	18 (86%)
Triceps	12 (57%)	9 (43%)	0 (0%)	5 (24%)	16 (76%)	5 (24%)	16 (76%)
Deltoid	13 (62%)	8 (38%)	0 (0%)	4 (19%)	17 (81%)	3 (14%)	18 (86%)

Abbreviations: EMG, electromyography; PSW, positive sharp wave potentials.

Note: PSW and fibrillations are spontaneous depolarization of denervated muscle fiber(s) indicative of axonal injury. Recruitment is the activation of successive motor units to increase the force of voluntary muscle contraction.

## Operative Technique

**Fig. 2:**
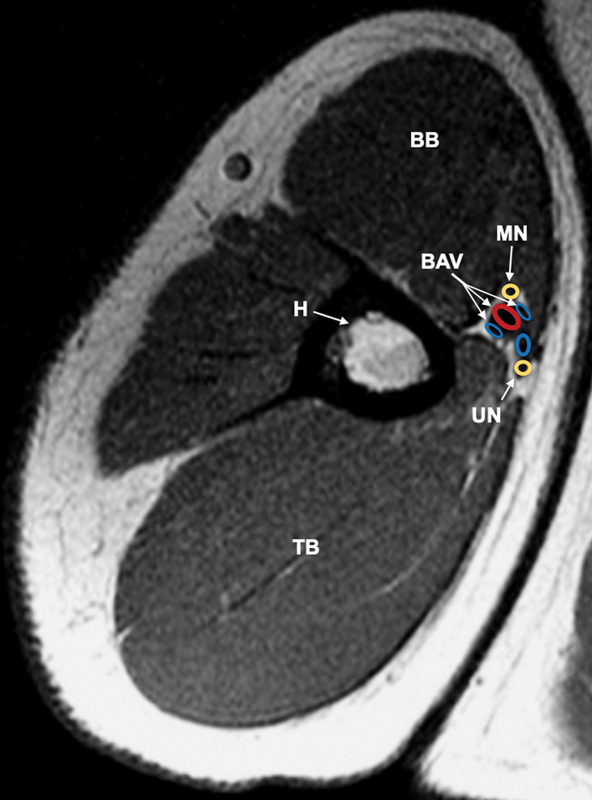
MRI ( *axial view* ) of the upper extremity illustrating structures within the MBFC. BAV, brachial artery and veins; BB, biceps brachii; BV, basilica vein; H, humerus; MN, median nerve; MBFC, medial brachial fascial compartment; MRI, magnetic resonance imaging; TB, triceps brachii; UN, ulnar nerve.

**Fig. 3:**
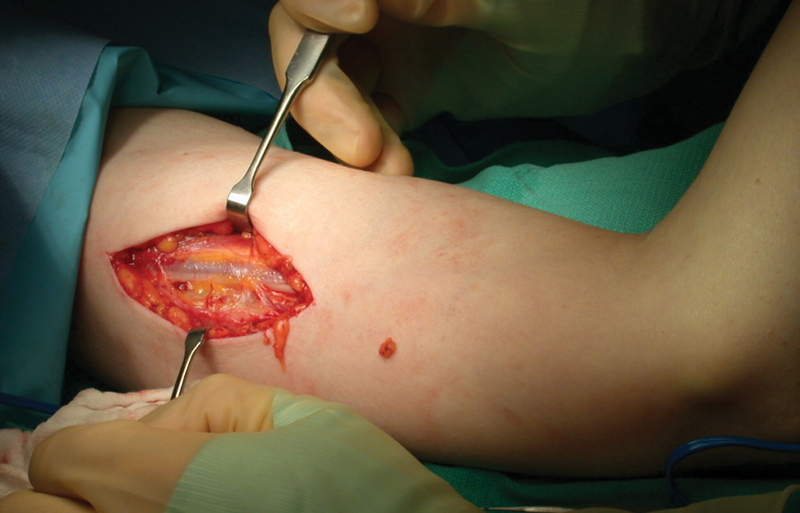
Longitudinal incision along the medial bicipital groove. Subcutaneous tissue is retracted exposing the underlying medial brachial fascial compartment.

**Fig. 4:**
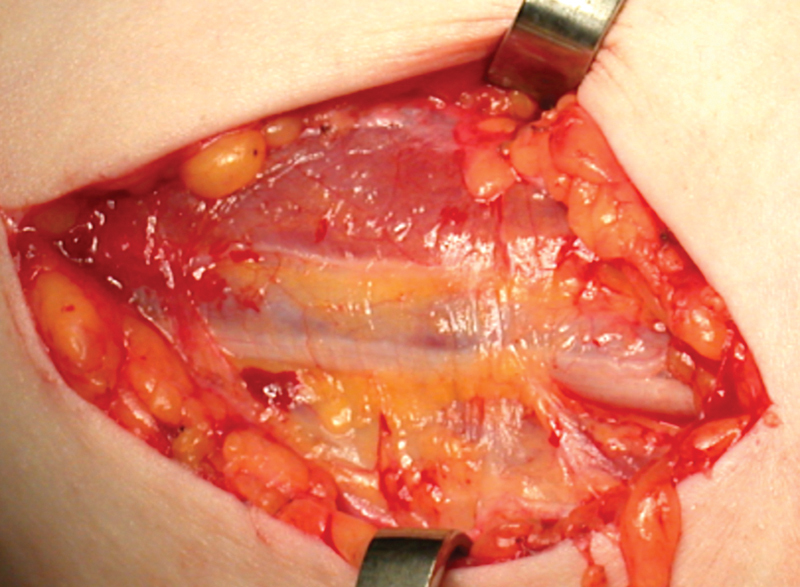
Connective tissue that comprises the MBFC and encloses the neurovascular bundle. MBFC, medial brachial fascial compartment.

**Fig. 5:**
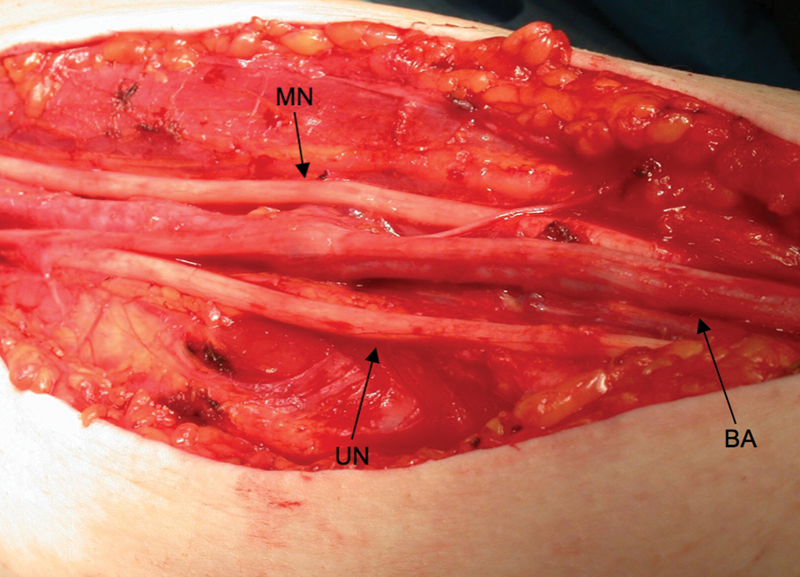
Neurovascular elements are identified and released. Fascia is excised and adhesions are lysed. Nerves and vessels lay loosely in a healthy tissue bed. BA, brachial artery; MN, median nerve; UN, ulnar nerve.

The anatomy of the MBFC is illustrated in Fig. 2 . All procedures were performed by the senior author and the same technique was employed for each patient. Patients were oriented in a beach chair position then prepped and draped in a sterile fashion. They underwent general anesthesia induction and subsequent IV Bier block that consisted of lidocaine and dexmedetomidine. A single longitudinal transcutaneous incision was initiated just distal to the intersection of the pectoralis major and short head of the biceps brachii. The incision was continued distally along the medial bicipital groove, in line with the humerus, approximately 10 cm. Connective tissue surrounding the underlying neurovascular bundle was dissected and the ulnar nerve, median nerve, brachial artery and vein, and basilic vein were identified ( Fig. 3 ). The dissection was slow and tedious to protect underlying structures. Thin connective tissue septa that extend inward from the outer sheath were resected. Adhesions formed within the compartment were lysed. All structures were carefully separated and arranged loosely within the tissue bed ( Fig. 4 ). Nerve continuity was grossly preserved in all cases ( Fig. 5 ). The procedure was considered complete when basilic vein dilation was visibly reduced, and all structures were decompressed. The site was assessed for hemostasis and subsequently sutured. We suggest using intraoperative nerve action potentials to aid in identification of brachial plexus structures during the procedure. Doppler ultrasound should be utilized to compare venous outflow before and after decompression. Patients were advised to wear an arm sling for 3 weeks. Physical and occupational therapies were started 2 weeks after surgery.

## Results

**Fig. 6:**
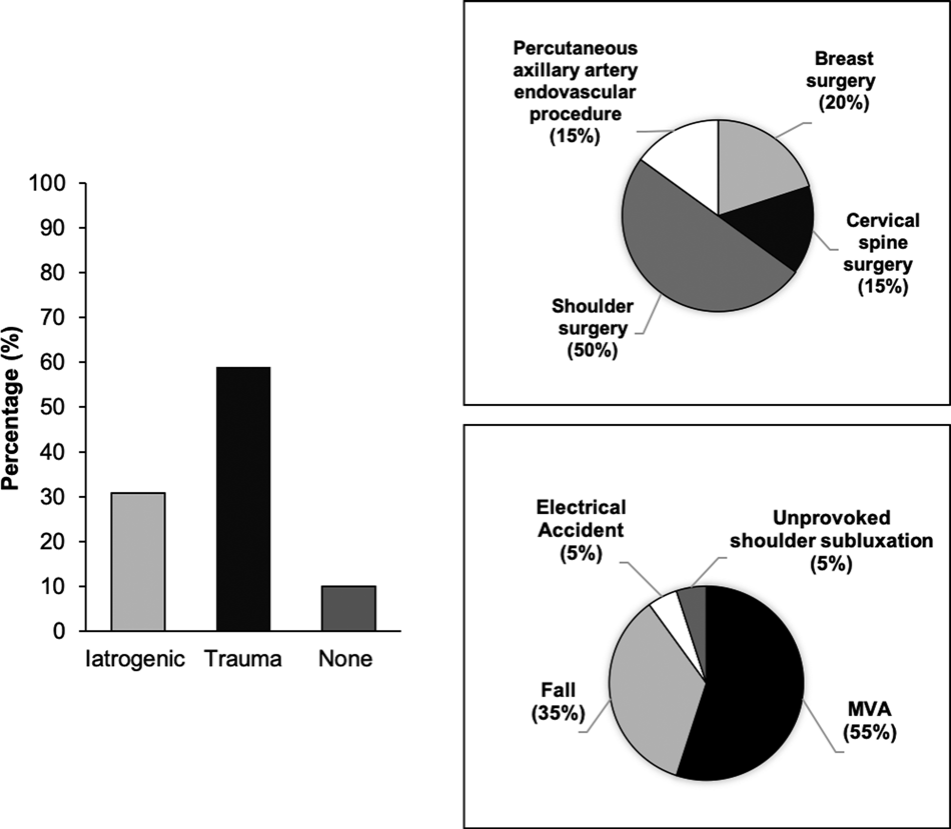
( **A** ) Etiology of brachial plexopathy in 21 patients who underwent surgical decompression in our clinic. ( **B** ) latrogenic causes further described. ( **C** ) Traumatic mechanisms represented in greater detail.

A total of 21 patients met inclusion criteria for this study. Table 5 summarizes the demographics of patients who underwent surgery. The mean age at the time of surgery was 56 years. Thirteen patients were female and eight were male. Most injuries involved the nondominant arm. Fig. 6 illustrates the mechanisms of injury. Fifty-nine percent of cases were attributed to trauma, 31% were iatrogenic, and 10% did not have a known cause. One patient fractured an arm and three patients dislocated their shoulder. Only one patient had a vascular injury to the upper arm. All patients in the present study had private health insurance.

**Table 5:** Demographics summary

Gender (M/F)	*n*	Male	Female
	21	8 (38%)	13 (62%)
Age (y)	*n*	Mean	Range
	21	56	22–80
Dominant arm (L/R)	*n*	Right	Left
	21	19 (91%)	2 (9%)
Injured arm (L/R)	*n*	Right	Left
	21	7 (33%)	14 (67%)
Interval	*n*	Median	Range
Symptoms onset to surgery (mo)	21	11	2–103
Surgery to post-op evaluation (≥6 mo)	21	10	6–85

Abbreviations: M, male; F, female; y, years; L, left; R, right; mo, months.

The median interval from symptoms onset to surgery was 11 months and 10 months from surgery to postoperative follow-up evaluation (>6 months). At the time of the procedure, the majority of patients underwent concurrent nerve release distally in the arm. Nerve decompression was performed at the cubital tunnel and carpal tunnel in 40% and 49% of patients, respectively.

**Fig. 7:**
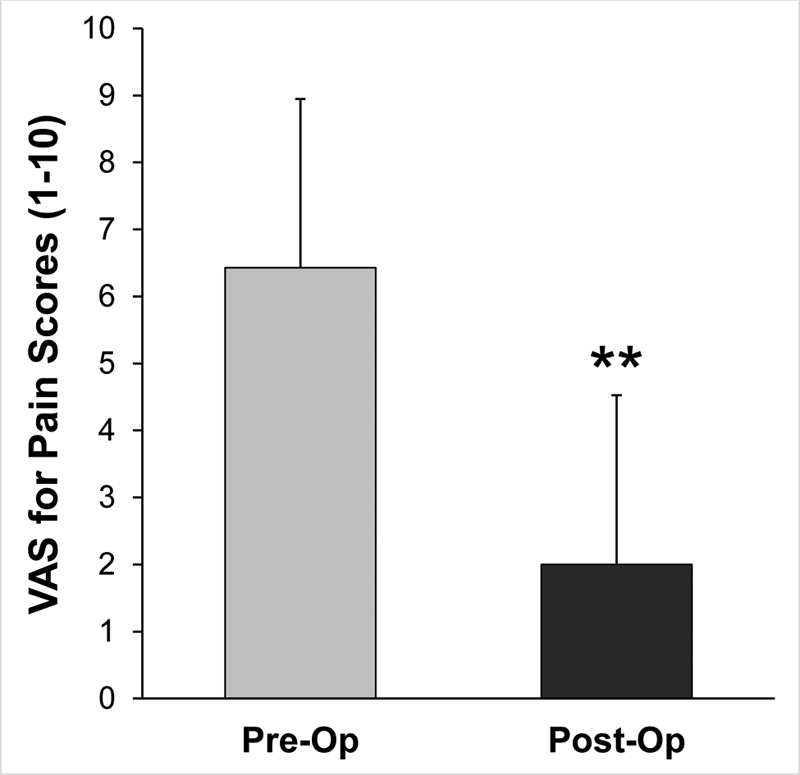
Visual analog scale (VAS) for pain scores within the brachium of the affected extremity, before and after surgery. Data shown as mean ± SD. ( *n* = 21 patients). * *p* < 0.05, ** *p* < 0.01. SD, standard deviation.

The VAS pain score was documented in the EMR for 21 patients, before and after surgery. A statistically significant reduction in VAS score was found when preoperative and postoperative values were compared. The mean preoperative VAS score was 6.4 (standard deviation or SD 2.5) and the mean postoperative VAS score was 2.0 (SD 2.5). Fig. 7 shows the VAS pain score outcomes. Pain was reduced by at least 30% in 14 of the 21 patients.

**Fig. 8:**
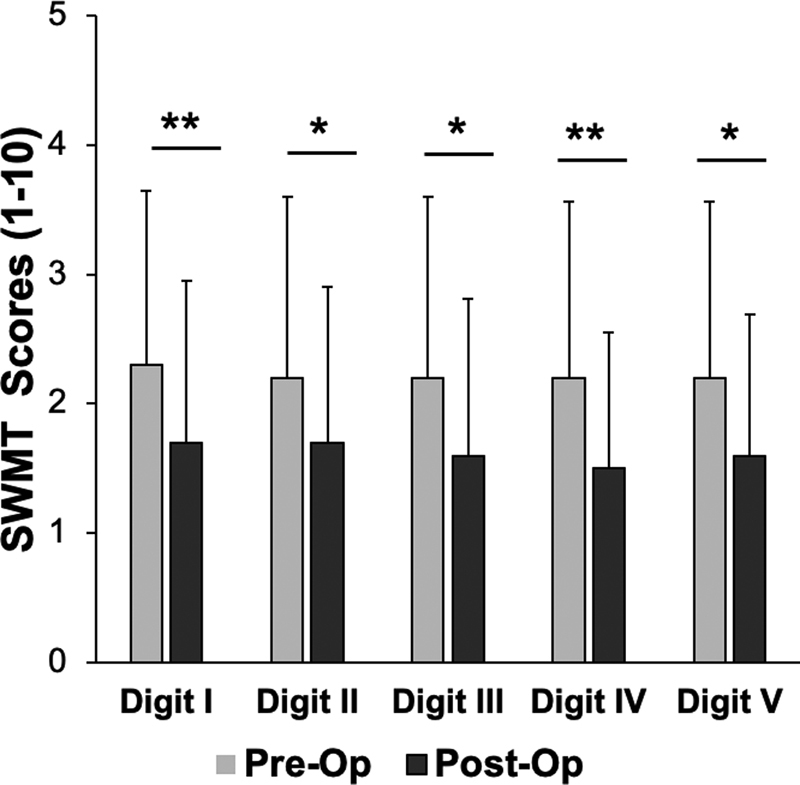
Semmes-Weinstein monofilament test (SWMT) for tactile sensation within the finger pulps of the affected extremity, before and after surgery. Data shown as mean ± SD. ( *n* = 21 patients). * *p* < 0.05, ** *p* < 0.01. SD, standard deviation.

The SWMT score was analyzed for 21 patients, before and after surgery. SWMT is graded on a 1 to 5 scale and the score is interpreted as (1) normal, (2) diminished light touch, (3) diminished protective sensation, (4) loss of protective sensation, and (5) responsive only to deep pressure sensation. As shown in Fig. 8 , SWMT scores were lower after surgery in each finger of the affected extremity; digit I (2.3 ± 1.4 vs. 1.7 ± 1.2), digit II (2.2 ± 1.4 vs. 1.7 ± 1.2), digit III (2.2 ± 1.4 vs. 1.6 ± 1.2), digit IV (2.2 ± 1.4 vs. 1.5 ± 1.1), digit V (2.2 ± 1.4 vs. 1.6 ± 1.1). Statistically significant lower sensory detection thresholds were achieved in digit I ( *p* = 0.01), digit II ( *p* = 0.03), digit III ( *p* = 0.02), digit IV ( *p* = 0.01), and digit V ( *p* = 0.02).

Table 6 summarizes MRC muscle scale grades, before and after surgery. More patients demonstrated an MRC grade ≥3 and ≥4 after surgery in elbow flexion, finger abduction, PIP/DIP extension, and thumb abduction. There was a 23% increase in patients who exhibited an MRC ≥4 in elbow flexion after surgery. Notably, elbow flexion is the most important indication of motor recovery in brachial plexus injury.

**Table 6:** Summary of Medical Research Council (MRC) scale for muscle strength grades within the affected extremity

		Pre-op		Post-op	
Active movement	*n*	MRC ≥ 3	MRC ≥ 4	MRC ≥ 3	MRC ≥ 4
Elbow flexion	21	18 (86%)	12 (57%)	20 (93%)	18 (86%)
Finger abduction	21	16 (76%)	13 (62%)	17 (81%)	15 (71%)
PIP/DIP extension	21	17 (81%)	16 (76%)	18 (86%)	17 (81%)
Thumb abduction	21	17 (81%)	13 (62%)	18 (86%)	17 (81%)

Abbreviations: DIP, distal interphalangeal joint; PIP, proximal interphalangeal joint.

## Discussion

Each case of brachial plexopathy consists of a unique pattern of Sedon and Sunderland classifications of nerve injury. Despite the heterogenous nature of brachial plexus injury, a commonality among these patients is pain in the affected extremity. Pain stemming from nerve compression (often used interchangeably with “entrapment”) is particularly impervious to conservative management. 25 The majority of nerve-in-continuity lesions after trauma, due to compression by scar tissue, occur at level IV of the brachial plexus. 26 At this level of the brachial plexus, terminal nerve branches course through the MBFC in close proximity to vascular and bony structures. The tight compartmental space and rigid surrounding connective tissue predispose underlying nerves to a compression neuropathy, which may be triggered after trauma to the brachium. The extent of nerve injury from entrapment can be measured using neurodiagnostic testing: commonly demonstrating reduced conduction velocity, prolonged latency, decreased sensory nerve action potential and compound muscle action potential amplitudes, fibrillation potentials and positive sharp wave potentials, decreased motor unit recruitment and giant motor unit potentials. 13 27 In our clinic, percussion tenderness over the proximal medial bicipital group was a valuable clinical finding in patients with compression neuropathy originating within the MBFC. Open fasciotomy and external neurolysis were performed in 45 patients with intractable symptoms. We studied the surgical outcomes of 21 patients who met criteria.

A 47% reduction in pain was observed in patients who underwent open fasciotomy and external neurolysis at level IV of the brachial plexus. Prior work has demonstrated that a 30% decrease in pain intensity is a clinically significant treatment response. 28 Overall, 67% of patients in our clinic had clinically relevant pain relief after surgery. Surgical management of neuropathic pain is particularly useful when the source is properly identified, nerve continuity is preserved, and the most appropriate procedure is employed. Preoperative neurodiagnostic findings demonstrated slow conduction velocity and decreased amplitude in the distal parts of the brachial plexus. Our findings corresponded with a pattern of demyelination and axonal dropout that signified chronic compressive neuropathy. External neurolysis was selected because nerve continuity was preserved throughout the brachial plexus in all patients. Surgical exploration revealed dense cicatricial tissue, or scar tissue, involving nerves and vasculature within the compartment. Compression, strangulation, and tethering of the nerve(s) by cicatricial tissue induce hypernociception by locally upregulating neurotrophic and inflammatory mediators. 29 30 31 External neurolysis liberates the nerve(s) from surrounding scar tissue, thereby, promoting an environment that is amenable to structural and functional nerve recovery and, ultimately, attenuation of hypernociception.

Excellent pain outcomes have been demonstrated from external neurolysis at common sites of compression of the median, ulnar, and lateral femoral cutaneous nerves. Narakas found adequate pain alleviation after neurolysis of intraneural and extraneural fibrosis throughout the entire brachial plexus. 32 Millesi demonstrated complete pain resolution in a brachial plexus injured patient who underwent neurolysis 2 years after an accident. 33 Several authors have argued that neurolysis may be beneficial but to a lesser degree than demonstrated in other studies. 34 35 Despite its relative safety and proven success with pain outcomes, external neurolysis continues to be understated as an option for level IV injuries. For the first time, we demonstrate the benefits of pan-plexus level IV decompression using external neurolysis within the medial brachial plexus fascial compartment.

The MBFC spans from the neck to the proximal arm and consists of fibrous connective tissue that surrounds blood vessels and nerves of the brachial plexus. Within the compartment, there is loose connective tissue and cleavage planes that allow for longitudinal excursion of each nerve. In a study on cadavers with an unknown history of trauma, anatomical dissections of the brachial plexus sheath frequently unveiled scattered scar tissue throughout the semirigid compartment space. 36 The presence of scar tissue may be trauma-induced or related to “natural wear and tear” in an otherwise healthy arm. It is generally accepted that a localized fibrotic reaction develops over the course of weeks to months after trauma, causing extensive damage to the supportive connective tissue. 37 In good agreement with the epidemiological literature on brachial plexus injuries, traumatic events were the most common etiology of nerve lesions among our patients. Although there is intriguing evidence that points to increased occurrences of peripheral nerve entrapment after orthopaedic trauma, only 4 of the 21 patients in the present study sustained a joint dislocation or bone fracture to the upper arm on imaging. Thus, fractures were unlikely to be a direct cause of nerve damage in these cases. We speculate that the gradual aggregation of intraneural and extraneural scar tissue, edema, and hyperemia within the MBFC led to the worsening of symptoms. As post-traumatic extraneural scar tissue is maintained over time, it directly gives rise to nerve dysfunction by restricting longitudinal excursion and propelling ischemic and mechanical damage. 38 The scar tissue also constricts venous return, which increases extraneural edema. The accumulation of extraneural edema elevates intracompartmental pressure and causes additional nerve damage. 39 In sum, the structural and functional changes induced by local physiological changes after trauma contribute to compression-induced nerve dysfunction.

The histological changes commonly observed in chronic compression neuropathy include: intrafascicular edema, undulant demyelination and remyelination, altered myelin thickness, abnormal internodal length, apoptosis and proliferation of Schwann cells, altered expression of receptors and channels, as well as perineural and epineural fibrosis. 38 39 40 41 42 43 44 45 46 47 48 Interestingly, it has been shown that proximal compression predisposes the nerve to secondary injuries at distal sites as it courses through tight anatomical spaces. The mechanism behind this theory, known as the “double crush syndrome,” is yet to be fully elucidated but is thought to involve impaired anterograde axonal transport of neuronotrophic factors essential to regulating neuronal functions. 49 50 51 52 Disturbed transportation of these key substances may induce aberrant morphological and functional alterations at distal sites. Likewise, compression at a distal site may lend the proximal nerve vulnerable to compression injury secondary to impaired retrograde transport according to the “reversed double crush syndrome.” 52 As these theories pertain to findings in the present study, nearly half of our patients had carpal tunnel syndrome and/or cubital tunnel syndrome. We believe that simple decompression and external neurolysis at one site bolstered recovery at the other.

Long-standing nerve compression does not preclude the ability for intrinsic repair if the impeding structure is adequately separated or removed with surgery. The timing of surgery may be key in nerve recovery but continues to be a topic of debate among experienced peripheral nerve surgeons, especially for certain techniques in nerve injury. The benefits of early intervention are twofold; to allow sufficient time for spontaneous nerve recovery and to prevent irreversible nerve damage. 53 There is general consensus that surgery should be considered in patients who have not demonstrated signs of spontaneous nerve recovery in 3 to 6 months. It has been suggested that optimal recovery occurs when surgery is performed 3 months after injury. 15 In spite of these well-defined recommendations, nerve-in-continuity lesions are amenable to surgery well after 6 months. In fact, Rochkind and Alon demonstrated successful return of function in patients who underwent neurolysis of the brachial plexus 1.2 to 12 years after injury. 54 In separate cases of neurolysis in late post-traumatic and ischemic neuropathies, Lusskin et al reported good sensory and motor recovery 9 years after injury. 55 Similarly, the benefits of neurolysis long after injury were observed in the present study. The median time to surgery was 11 months because many patients experienced delayed referral for surgical evaluation. Notwithstanding a prolonged duration since injury, functional recovery can be appreciated just after neurolysis in some cases. This improvement is due to surgical restoration of a microenvironment that previously constricted nerve function and recovery. In support, Swartz et al suggested that earlier than expected functional recovery after nerve grafting and transfers occurs with concomitant neurolysis. 56 They concluded that this finding was a result of nerve decompression from neurolysis rather than from spontaneous axonal regeneration and sprouting that occurs 6 months after injury. Conversely, several authors have suggested that neurolysis has a limited impact on functional outcomes, particularly when it is used as an isolated procedure.

Although the role of external neurolysis was previously thought to be limited to pain relief, more recent evidence indicates that it has a sizeable impact on sensory and motor function recovery. 26 32 57 58 59 60 61 Moreover, simple decompression and external neurolysis for nerve-in-continuity lesions have demonstrated much greater outcomes than any operative technique employed for other lesions of the brachial plexus. 58 In a study on nerve repair outcomes in traumatic brachial plexus injuries, Rasulić et al found useful functional recovery after neurolysis in 89.7% of all cases; including the axillary nerve (100%), median nerve (100%), radial nerve (84%), and ulnar nerve (69.2%) terminal branches. 18 At level IV brachial plexus injuries, Lam reported good motor outcomes after neurolysis in 12 of 13 patients within a year after surgery. Matejcik and Penzesova reported on 59 patients who underwent neurolysis of the brachial plexus, in which, 25 regained complete mobility and strength (MRC rating score = 5) and 52 attained notable improvements (MRC rating score = 3). 60 Younger age groups, particularly patients under 20 years old, have been shown to have greater functional outcomes after neurolysis. 62 In the present study, patients were comparatively more advanced in age (mean: 56 years) and still demonstrated impressive motor and sensory outcomes. Six of nine patients with a preoperative MRC rating score of 3 or less in elbow flexion clinically improved to a score of 4 or 5 after surgery. Improvements were also observed in motor function throughout the hand, although most patients had preserved strength in finger abduction, PIP/DIP extension and thumb abduction prior to surgery. Hand sensibility and prehension are priorities, behind elbow flexion, in surgical repair strategies of the brachial plexus. Sensory recovery in the hand is integral to fine manipulation needed to perform occupational or routine skills. Overall, there were significant improvements in perception to light touch after surgery in the 21 patients tested. Interestingly, a close association between sensory recovery in the hand and pain relief has been reported in the past.

An open surgical approach was used for all cases in this study. The importance of wide-exposure in compression-induced nerve lesions has been emphasized in the past. The brachial plexus is technically difficult to navigate during surgery because of the complex and intricate network nerves entangled with great vessels and connective tissue. Additionally, there is considerable anatomical variability within the MBFC that requires open exploration to comprehensively address each unique case. It is thought that minimally invasive techniques do not offer adequate visibility needed to engage all causative abnormalities within the distorted anatomical space. 63 Furthermore, Lusskin et al advised of the potential risk of nerve devascularization during neurolysis. 55 Wide exposure may help protect the nerve and prevent surgical revision(s). Each revision may amplify local scar density, worsening nerve compression that manifests as neuropathic pain. Indeed, the recurrence of pain several years after complete pain resolution from neurolysis has been reported and may be linked to the postoperative compilation of cicatricial tissue. 37 Long-term follow-up would be required to assess the rate of recurrence in our patients. Although an open approach was preferred in the present study, there have been promising advances in endoscopic operative techniques for brachial plexus exploration. Current literature assessing visibility in endoscopic approaches has reported mixed results. 64 65 66 67 Even though an endoscopic operative technique is plausible for supraclavicular repairs, it is less practical for level IV injuries of the terminal branches. There may be a higher risk of nerve injury while using an endoscopic technique for nerve decompression. Apart from that, open and endoscopic approaches have demonstrated similar long-term pain and functional outcomes when used for nerve decompression. 68

## Limitations

Future work should incorporate postoperative neurodiagnostic findings to quantify changes to electrical activity after surgery. Post-surgical EMG/NCS were not indicated in these cases and it is likely that most patients would have refused these studies. Thus, we were unable to objectively measure the neurophysiological effects of surgery as it pertains to changes in nerve structure and function. Socioeconomic and demographic factors may have impacted our data. All patients had private health care insurance and our results may not be reflected in an underserved population. Also, the majority of our patients were female even though a higher prevalence of brachial plexus injury has been established in males. A predilection of women to be treated by a female (the corresponding author) may have contributed to this finding. A prospective study using a handheld dynameter to quantify motor strength more precisely may also provide stronger data to support the present findings. The HSS questionnaire to measure impact of brachial plexus injury and assess surgical outcomes is a promising tool that will be used in our future research.

## Conclusion

The MBFC is prone to accumulating of scar tissue after trauma. Over time, the scar tissue can involve nerves of the brachial plexus resulting in a compression-induced dysfunction. We demonstrated successful pain, motor and sensory outcomes after external neurolysis for nerve-in-continuity lesions within the MBFC. Future investigations may further elucidate the benefits of this procedure when used in conjunction with distal nerve release. Our results support the utility of external neurolysis in patients with pain and nerve-in-continuity lesions of the terminal branches.
